# Comparative Morphology of the Wing Base Structure Illuminates Higher-Level Phylogeny of Holometabola

**DOI:** 10.3390/insects15030199

**Published:** 2024-03-16

**Authors:** Chenjing Zhao, Mengting Huang, Ding Yang, Xingyue Liu

**Affiliations:** 1 Department of Biology, Taiyuan Normal University, Jinzhong 030619, China; zhaochenjing398@126.com (C.Z.); 18378229937@163.com (M.H.); 2 Department of Entomology, China Agricultural University, Beijing 100193, China

**Keywords:** Holometabola, wing base, cladistic analyses, morphological study

## Abstract

**Simple Summary:**

Insects that undergo complete metamorphosis, collectively known as Holometabola, are the most successful lineage of living organism, including about 830,000 species. Understanding the intricate relationships among the major groups of holometabolous insects is a critical task in systematic biology, particularly given their immense diversity. This research analyzed the wing base structure of Holometabola using comparative morphology to further clarify several crucial relationship nodes within Holometabola. Morphological data were selected from both the forewing base and hindwing base, comprising fifty-three discrete characters. Many phylogenetic relationship nodes among Holometabola were recovered exclusively using the wing base structure. Our research further highlights the efficacy of wing base morphology data in understanding insect phylogeny and evolution.

**Abstract:**

Phylogenetic relationships among Holometabola have been the subject of controversy. The value of the wing base structure in phylogenetic analysis has been demonstrated but remains largely underexplored and scarce in studies of Holometabola. We studied the phylogenetic relationships among Holometabola (excluding Siphonaptera), focusing exclusively on wing base structure. Cladistic assessments were conducted using 53 morphological data points derived from the bases of both the forewing and hindwing. The results of wing base data revealed a sister relationship between Hymenoptera and remaining orders. The sister-group relationships between Strepsiptera and Coleoptera, Mecoptera and Diptera, Trichoptera and Lepidoptera, and Neuropterida and Coleopterida were corroborated. In Neuropterida, our results recovered the sister relationship between Megaloptera and Neuroptera, as well as the monophyly of Megaloptera.

## 1. Introduction

Holometabola is the most abundant group in the insect class, so far, with about 830,000 species having been described, comprising more than 80% of all described metazoan species and exhibiting substantial variation in ecology, behavior, and morphology [1,2]. This unprecedented diversity provides rich research materials and intricate morphological data for evolutionary biologists and phylogeneticists. Although much research has focused on group phylogeny, the relationships of some important nodes remain unresolved.

Holometabola has long been considered monophyletic and has never been seriously questioned. However, the internal relationships of this group are still a subject of debate. Hennig was the pioneer in offering the first extensive reconstruction of the phylogenetic relationships within Holometabola [3]. The basal phylogeny in his proposal is represented as (Coleoptera + Neuropterida) + (Hymenoptera + Mecopterida). Hymenoptera were placed as the sister group of Mecopterida, a relationship that was confirmed by whole mitochondrial genomes and early morphological data [4,5,6]. This result, however, was overturned by numerous studies based on whole nuclear or EST datasets, which overwhelmingly supported Hymenoptera as the sister group of the remaining Holometabola [7,8,9,10,11]. The remaining Holometabola are typically divided into two major lineages, as follows: Mecopterida (Amphiesmenoptera + Antliophora (including Strepsiptera?) and Neuropteroidea (Coleoptera (+Strepsiptera?) + Neuropterida)) [6,12,13,14,15]. In recent years, phylogenetic studies based on nuclear protein coding genes and morphological evidence have consistently confirmed the basal position of Hymenoptera, thereby establishing this concept in a dominant position [16,17,18,19,20,21]. However, studies based on nuclear rRNA data usually obtain a wide variety of positions for Hymenoptera, for example, positioning them as sister groups with Neuropterida, which contributes to the persisting uncertainty regarding the placement of Hymenoptera [22,23,24,25].

The position of Strepsiptera in the phylogeny of Holometabola is another highly disputed issue due to their remarkably derived lifestyles and morphological characters. Their peculiar phenotype, characterized by conspicuous sexual dimorphism, shows that females are strongly morphologically simplified, with a complete lack of legs, wings, and external genitalia. This group has shown fascinating endoparasite behavior, with the diminutive primary larvae parasitizing other insects. In the past few decades, extensive studies have placed Strepsiptera in different positions [26]. Traditionally, morphological characters uniting Strepsiptera and Coleoptera based on a broad array of morphological features include different body regions and unusual life stages [14,16,17]. In contrast, a study integrating molecular and morphological data proposed a clade comprising Strepsiptera and Diptera (Halteria). This was based on ribosomal DNA sequence parsimony analyses and the proposed homology between the hind halteres of flies and the fore halteres of Strepsiptera [13]. Alternative placements of Strepsiptera have also been proposed; these include positioning Strepsiptera either as a sister group of Neuropterida or as derived from within the order Coleoptera [19]. Recently, the theory proposing a sister-group relationship between Strepsiptera and Coleoptera has gained more credibility from phylogenetic studies using nucleotide sequences obtained from comprehensive genome sequencing projects [27]. However, a new study failed to confirm the sister-group relationship between Coleoptera and Strepsiptera [28]. In conclusion, the phylogenetic position of Strepsiptera within Holometabola remains ambiguous due to such inconsistent research results.

Mecopterida is the largest lineage within Holometabola, comprising two clades, namely Amphiesmenoptera (Lepidoptera + Trichoptera) and Antliophora (Diptera + Siphonaptera + Mecoptera). The relationships within Antliophora and the monophyly of Mecopterida have been controversial [29]. The monophyly of Mecopterida was found by Wiegmann et al. [21], while Kukalová-Peck et al. and Beutel et al. failed to find this concept in their analyses [11,17]. Based on molecular evidence, Whiting suggests that Mecoptera and Siphonaptera are paraphyletic, while other orders are monophyletic [30]. Wiegmann et al. refuted this hypothesis by analyzing several single-copy protein-coding nuclear genes and recovered the monophyly of Mecoptera [21]. Therefore, the monophyly and the relationships within Mecoptera have been a subject of debate. Recently, Meusemann et al. attempted to clarify the phylogeny of Antliophora and verify the monophyly of Mecoptera by analyzing extensive transcriptomic nucleotide sequence data [29]. However, their data did not lead to a definite conclusion. Cai et al. attempted to resolve the phylogenetic controversy within the diverse and medically significant group of fleas [31]. They used the most extensive molecular dataset to date, comprising over 1400 protein-coding genes. Fleas were consistently identified as nested within scorpionflies (Mecoptera), forming a sister relationship with Nannochoristidae. Zhang et al. reconstructed the phylogeny of fleas among Endopterygota using mitochondrial phylogenomics of two species orders [32]. They provided positive support for the hypothesis that Siphonaptera are monophyletic and demonstrated a sister-group relationship between Siphonaptera with orders Diptera + Mecoptera + Megaloptera + Neuroptera. In summary, conflicting phylogenetic relationships among Mecopterida need further clarification.

Within Neuropteroidea (Neuropterida, Coleoptera, and Strepsiptera), major phylogenetic confusion is focused on the monophyly of this group and the relationships among Neuropterida. Neuropterida have been confirmed as monophyletic by some morphological data [21,33] and several recent molecular studies [1,34,35]. This hypothesis, in contrast, was disapproved by Kukalová-Peck [11] and Beutel et al. [17]. Longstanding, competing hypotheses have been proposed with respect to Neuropterida (Neuroptera, Raphidioptera, and Megaloptera), archaic holometabolous insects, and the inter-relationships of their three orders, possibly due to enormous disparities in morphology and lifestyle. Numerous studies indicate that Megaloptera are monophyletic [1,15,35,36,37]. However, Beutel et al. [17] proposed a clade comprising Raphidioptera + Corydalidae based on analysis of the morphological character set and molecular data, a finding consistent with the work of Wiegmann et al. [21] and Winterton et al. [38]. With respect to the inter-relationships within Neuropterida, which are traditionally based on morphological data, a sister-group relationship between Megaloptera and Raphidioptera has been hypothesized [12,17]. However, another cladistic analysis of morphological characters presented a sister-group relationship between Megaloptera and Neuroptera, as proposed by Aspöck [39]. Recently, Song et al. [1] used mitogenomic data to identify a clade that includes Neuroptera and a sister group comprising Megaloptera and Raphidioptera. This finding supports previous hypotheses about the close relationship between Megaloptera and Raphidioptera. To reconcile these phylogenetic contradictions, a substantial amount of molecular evidence [35,40,41,42,43,44,45,46] and morphological data [2,47,48,49] have been presented, leading to the recovery of the monophyly of Megaloptera and the sister-group relationship between Megaloptera and Neuroptera.

Since the prosperity of molecular systematics, insect morphology has suffered a decline after flourishing in the first two-thirds of the 20th century [50]. Innovative molecular techniques offer the outstanding capability to address many phylogenetic questions that are difficult to solve with morphological methods and provide the ability to generate large-scale genomic datasets in a comparatively short time. However, in the latest study of Neuropterida, the authors analyzed a vast amount of molecular data but found that certain nodes in the Neuroptera tree were not robustly resolved. Consequently, they advocate for integrating morphological analyses with sequence-based phylogenomic data [46]. Arguably, such controversial hypotheses provided a healthy adjustment and forced morphologists to re-evaluate time-honored hypotheses and generate new morphological data. It is conceivable that the production of abundant morphological records and comprehensive combined datasets is necessary to further advance insect phylogenetics [13,46,51,52].

The evolution of foldable wings marks a seminal milestone in insect diversity, endowing them with superior mobility for migration, feeding, and so on. The intricate systems of elegant insect wings include membranes, veins, folding and flexion lines, and marginal setae. All these elements work together in a way that we understand only partially [53]. A cluster of inter-related sclerites at the wing’s base, where it connects to the thorax, is essential for various wing actions, including flapping, rotation, and folding. Given their critical functional role, these sclerites exhibit minimal evolutionary change, reflecting their significant mechanical constraints [47,54]. In addition, the intricate shapes and articulations of the wing base sclerites make it possible to provide more important morphological characters for phylogenetic estimations [6,47,54,55,56,57,58]. Furthermore, the phylogenetic trees based on wing base morphology align with findings from molecular phylogenetic studies and comprehensive insect phylogenomic research [47]. Currently, research on wing base sclerites is predominantly focused on hemimetabolous insects. For instance, the morphology of wing base sclerites of Polyneoptera and Paraneoptera has been extensively explored, which also applies to the studies of phylogenetics and evolution [59]. In contrast, research into the wing bases of holometabolous insects remains relatively limited. Here, we provide a morphological data matrix based on wing base sclerites focused specifically on the phylogeny of Holometabola. The main objective of this study is to develop a detailed and thoroughly documented set of morphological characters, focusing on the structures of the forewing and hindwing bases. The character states were analyzed using heuristic parsimony analysis. Our investigations also provide new phylogenetically relevant data for understanding of the higher phylogeny of Holometabola.

## 2. Materials and Methods

### 2.1. Examined Taxa

Ingroup taxa included the species representing three families/subfamilies of Megaloptera, three families of Neuroptera, two families of Raphidioptera, five families of Coleoptera, three families of Hymenoptera, one family of Trichoptera, two families of Lepidoptera, two families of Mecoptera, four families of Diptera, and one family of Strepsiptera (Table S1). For this experiment, research materials were selected from different orders, including Xyelidae, Tenthredinidae, and Diprionidae in Hymenoptera; Nevrorthidae, Osmylidae, and Chrysopidae in Neuroptera; Corydalidae and Sialidae in Megaloptera; Raphidiidae and Inocelliidae in Raphidioptera; Cupedidae, Carabidae, Cicindelidae, Cerambycidae, and Melolonthidae in Coleoptera; Tipulidae, Pyrgotidae, Syrphidae, Tabanidae, and Phryganeidae in Diptera; Phryganeidae in Trichoptera; Corioxenidae in Strepsiptera; Sphingidae and Nymphalidae in Lepidoptera; and Bittacidae and Panorpidae in Mecoptera. The outgroup selection included Gripopterygidae of Plecoptera and Tettigoniidae of Orthoptera, both of which belong to polyneopterous, a group that is a sister clade to the paraneopteran + Holometabola [12]. The wing base structure was observed using a ZEISS Stemi 2000-c stereoscope (Carl Zeiss, Jena, Germany). All of the wings were stretched artificially upwards during observation to account for their highly three-dimensional structures. Additionally, all figures were taken from the dorsal view of the wing base. Upon collection, all examined specimens were first stored in 80% ethanol, then moved to long-term storage at −20 °C at the Entomological Museum of China Agricultural University (CAU) in Beijing.

### 2.2. Terminology

The terminology for wing base sclerites is based on the works of Brodsky [60] and Matsuda [61], while folding line terminology is derived from Wootton [62]. In the text and illustrations, abbreviations like 1Ax–4Ax (representing the first to fourth axillary sclerites), ANWP, MNWP, PNWP (anterior, median, and posterior notal wing processes, respectively), pPNWP (pseudo-PNWP), and AmNWP (antemedian notal wing process), along with BA (basanale), BR (basiradiale), BSc (basisubcostale), HP (humeral plate), Tg (tegula), and PMP/DMP (proximal/distal median plates), are used.

### 2.3. Phylogenetic Analysis

Fifty-three characters from the fore- and hindwing bases were systematically coded for analysis, as detailed in the character description in the Results section. This coding only included quantitative aspects when variations were clearly distinguishable and not part of a continuous range. Although in most groups, the fore- and hindwing base structures usually exhibit analogous modifications [55,56,59,61], data are generally selected from the forewing or hindwing alone to prevent double counting of any particular character. However, in this study of Holometabola, several features of the hindwing base were markedly different from those of the forewing base. Hence, it was justified to select data from both the fore- and hindwings. 4Ax and PNWP are regarded as homologous sclerites, as discussed by Zhao et al. [47]. 4Ax is homologized with the pPNWP [57].

In the phylogenetic analysis, each family was considered as a terminal taxon within both ingroup and outgroup categories. We analyzed 26 forewing and 25 hindwing base characters using TNT ver. 1.1 [62] and NONA ver. 2.0 [63] and applied heuristic parsimony methods with 100 replications. Bootstrap analysis with 10,000 iterations was conducted, collapsing branches with values ≤ 50%. Bremer’s decay indices were calculated using TNT ver. 1.1 [62] and WinClada ver. 1.00.08 [64]. Detailed information about data matrix is shown in Table S2. In order to test, verify, and calculate the CI and RI of each character, the dataset was also analyzed in PAUP*4.0b10 [65]. This included a heuristic parsimony analysis with 100 random additions of taxa and TBR branch swapping, applying ACCTRAN optimization, treating characters as unordered and equally weighted, and using the MulTrees option.

## 3. Results

### 3.1. Comparative Morphology of the Wing Base Structures

**Figure 1:**
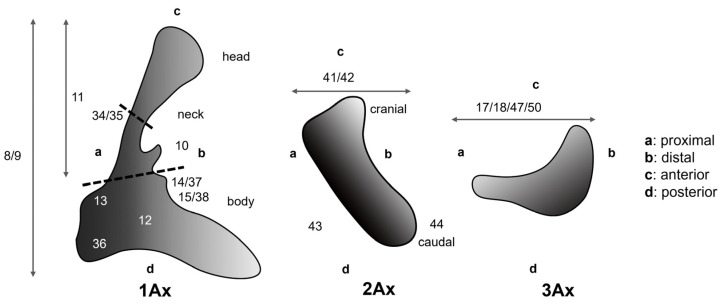
Overall model of the axillary sclerites for Holometabola: 1Ax, 2Ax, 3Ax. This model was modified from Figure 3 of Franielczyk-Pietyra et al. [66]. Numbers represent morphological characters. Detailed character descriptions are provided in the character description section.

The general morphology of the wing base structure was previously introduced and summarized by Zhao et al. [47]. Remarkably, several elements are particularly important in terms of function and phylogeny, notably the first and third axillaries [6]. Obviously, the second axillary plays a crucial supporting role in wing movement, but the actual transmission of the flight muscles’ actions from the notum to the wing primarily relies on the first axillary. The first axillary can be divided into the following three parts: the head, the neck, and the basal part (the body). According to Yoshizawa et al. [55], there is no obvious distinction between the head and the neck in Holometabola; however, in our study, we observed recognizable differences. The third axillary plays a critical role in Neoptera and in Holometabola, as it is equipped with a muscle that is considered an autapomorphy of Neoptera [6]. To facilitate the understanding of character descriptions, numerous characters are illustrated in schematic diagrams of the axillary sclerites, excluding the relationships among the axillaries. Each sclerite of the wing base is defined by proximal, distal, anterior, and posterior aspects (Figure 1) [66].

#### 3.1.1. Hymenoptera (Figure 2)

The wing base structure of Hymenoptera consists of fundamental elements. Articulations and fold and flexion lines also preserve the plesiomorphic condition. However, compared with other orders of Holometabola, the wing base structure of Hymenoptera is different. The first difference refers to the ANWP of the mesothorax. In Hymenoptera, the ANWP is consistently tubular and positioned anteriorly to the Tg in the forewing, whereas in other orders, the ANWP is triangular or stripe-like and located behind the Tg (char. 1, 2). In addition, 1Ax in Hymenoptera is distinguished from other groups by strong swelling (char. 9). In the anterodistal part of the body of 1Ax in Hymenoptera, there is a small projection that differs from projections of the neck of 1Ax based on the boundary between the neck and body of 1Ax (char. 14). Additionally, the 3Ax of Hymenoptera is obviously different from others; it is stripe-like with two lobes rather than plate-like with three lobes (char. 17, 18). The DMP appears more swollen and distinctly harder than the PMP and is smaller than the 1Ax in Hymenoptera (char. 22, 23). In other orders, 1Ax is usually smaller than the median plates. The shape of BA is similar to 3Ax, with a stripe-like character (char. 25). In the hindwing base, the neck of 1Ax is absent in Hymenoptera (char. 35). For 2Ax, its shape is almost rectangular and does not bend (char. 41). Meanwhile, in other groups, it is stripe-like and bends. In addition, the BSc and BR are completely fused in Hymenoptera (char. 46). In other orders, they are partly fused at most. In Hymenoptera, the BA is strong—larger than 3Ax in the hindwing (char. 53).

**Figure 2:**
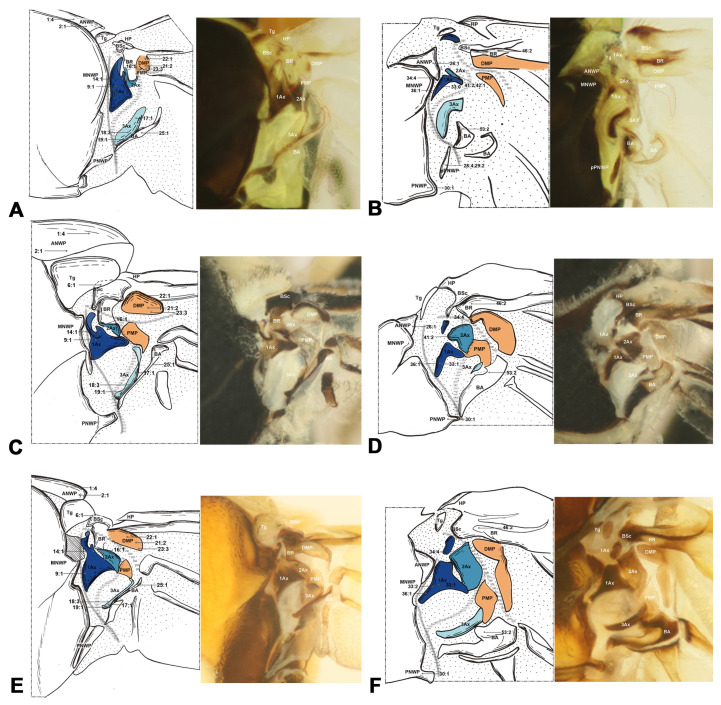
Wing base of Hymenoptera. The 1Ax is rendered dark blue, the 2Ax in medium blue, and the 3Ax in light blue. Both the PMP and DMP are illustrated in orange. (**A**) *Xyela* sp. (Xyelidae), forewing base; (**B**) same, hindwing base; (**C**) *Tenthredo* sp. (Tenthredinidae), forewing base; (**D**) same, hindwing base; (**E**) *Neodiprion huizeensis* Xiao & Zhou, 1984 (Diprionidae), forewing base; (**F**) same, hindwing base. Number of morphological characters: character state for phylogenetic analysis is indicated by a straight line for relevant position. Detailed character descriptions are provided in the character description section.

#### 3.1.2. Amphiesmenoptera (Figure 3)

Amphiesmenoptera contains Lepidoptera and Trichoptera. Compared to other Holometabola, the body of 1Ax in Amphiesmenoptera is more closely approximated to a rectangle, with the basal and distal lobes being of similar length (char. 12). In Lepidoptera, the Tg exhibits a triangular shape in the forewing and is absent in hindwing (char. 7, 31). The 1Ax tapers from the neck to the head in the forewing (char. 11). The 2Ax is stripe-like and articulates with 1Ax in the anterodistal part of the forewing (char. 39). The DMP integrates with BR into a sclerite in the forewing (char. 20). In Trichoptera, there is a projection in the anterodistal MNWP of the forewing, which might indicate a unique evolutionary characteristic of this order (char. 4).

**Figure 3:**
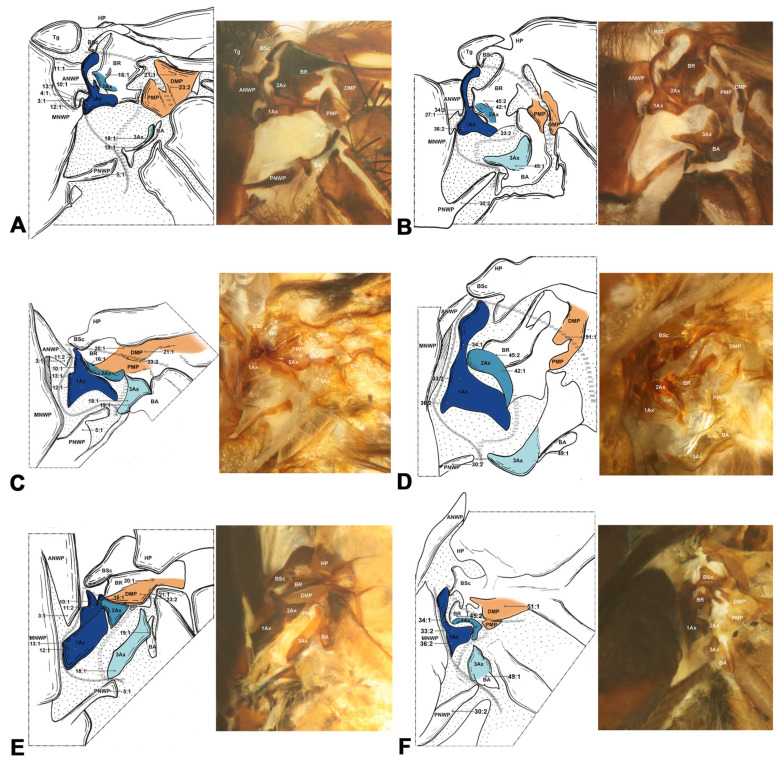
Wing base of Trichoptera (**A**,**B**) and Lepidoptera (**C**–**F**). (**A**) *Eubasilissa regina* (McLachlan, 1871) (Phryganeidae), forewing base; (**B**) same, hindwing base; (**C**) *Macroglossum* sp. (Sphingidae), forewing base; (**D**) same, hindwing base; (**E**) *Polygonia c-aureum* (Linnaeus) (Nymphalidae), forewing base; (**F**) same, hindwing base.

#### 3.1.3. Antliophora (Figure 4)

In Antliophora, the variation from the neck to the head of the 1Ax in the forewing is discontinuous, characterized by a narrowing at the neck–head boundary and a broadening at the apex of the head (char. 11). Another distinctive characteristic in Antliophora is that the BA is almost twice as large as the 3Ax in the forewing (char. 24). In Mecoptera, the structure at the wing base is composed of basic, essential elements. It was noted that the body of the first axillary presents a short and broad structure that is more rectangular than triangular in shape, typically featuring a concave caudal edge in both wings. This observation aligns with previous research, as discussed in the review by Hörnschemeyer [6]. We also noted that the Tg in Mecoptera is slimmer and exhibits a more rectangular shape compared to other species, where it tends to be more triangular (char. 7). Additionally, the shape of pPNWP is notably distinctive, exhibiting a U shape, while in Hymenoptera, it appears almost stripe-like (char. 28). In Diptera, the shape of the body of 1Ax similar to a lady’s high heels in the forewing (char. 12). In the hindwing, the width of neck is approximately the same as the head of 1Ax (char. 34).

**Figure 4:**
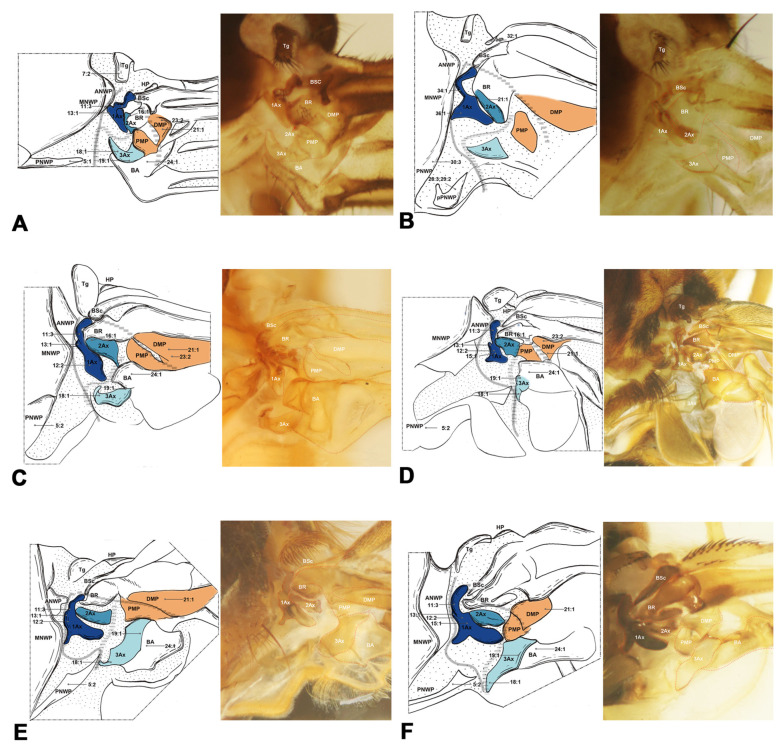
Wing base of Mecoptera (**A**,**B**) and Diptera (**C**–**F**). (**A**) *Bittacus* sp. Latreille (Bittacidae), forewing base; (**B**) same, hindwing base; (**C**) *Tipula (Nippotipula) sinica* Alexander (Tipulidae), forewing base; (**D**) *Tabanus* sp. (Tabanidae), forewing base; (**E**) *Apyrgota breviventris* Shi, 1998 (Pyrgotidae), forewing base; (**F**) *Episyrphus balteatus* (De Geer, 1776) (Syrphidae), forewing base.

#### 3.1.4. Coleopterida (Figure 5)

Coleopterida comprises Coleoptera and Strepsiptera. A notable characteristic in Coleopterida is that the DMP partially fuses with the PMP in the hindwing. In Coleoptera, the wing base comprises essential, foundational elements, but lacks the Tg, which is mainly an autapomorphy for this order (char. 6). A unique and fascinating feature in the wing structure is the presence of a small independent sclerite, located between the first and third axillary in the membrane, which we consider to belong to 3Ax. This sclerite may serve as a muscle attachment point in Coleopterida, differing from other Holometabola, where muscles are directly attached to the third axillary (char. 47). The metanotum is longer than the 1Ax, being about 1.4 to 2.4 times the length of the 1Ax (char. 33). In our study of Strepsiptera, a notable feature observed in our study is the absence or unrecognizability of the HP in the hindwing (char. 32).

**Figure 5:**
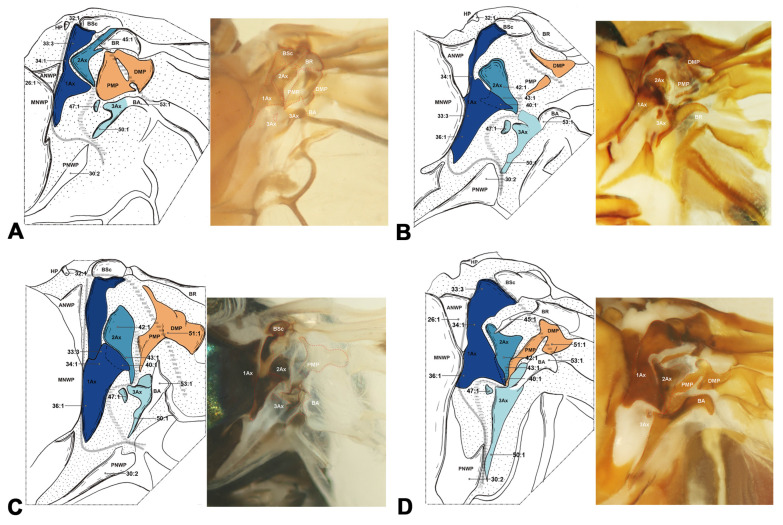
Wing base of Coleoptera. (**A**) *Tenomerga* sp. (Cupedidae), hindwing base; (**B**) *Chlaenius* sp. (Carabidae), hindwing base; (**C**) *Cylindera* sp. (Cicindelidae), hindwing base; (**D**) *Trichoferus guerryi* (Pic, 1915) (Cerambycidae), hindwing base.

#### 3.1.5. Neuropterida (Figure 6, Figure 7 and Figure 8)

In Neuropterida, the wing base structure includes essential elements, with its articulations, as well as fold and flexion lines, reflecting ancestral features. In Neuropterida, the pPNWP of the metathorax is sclerotized rather than membranous or absent in other clades of Holometabola (char. 29). The shape of the 2Ax is almost stripe-like, but it bends distally at the apex in this clade (char. 41). In addition, in the hindwings of Neuropterida, the BSc and BR are partly fused, in contrast to Hymenoptera where they are completely fused in the hindwings (char. 46). The 3Ax in Neuropterida consists of three lobes, the anterior, proximal, and distal lobes (char. 17). A separate part is present in the PNWP of the hindwing. Further details about the wing base structure in Neuropterida are described by Zhao et al. [47], without additional discussion here.

**Figure 6:**
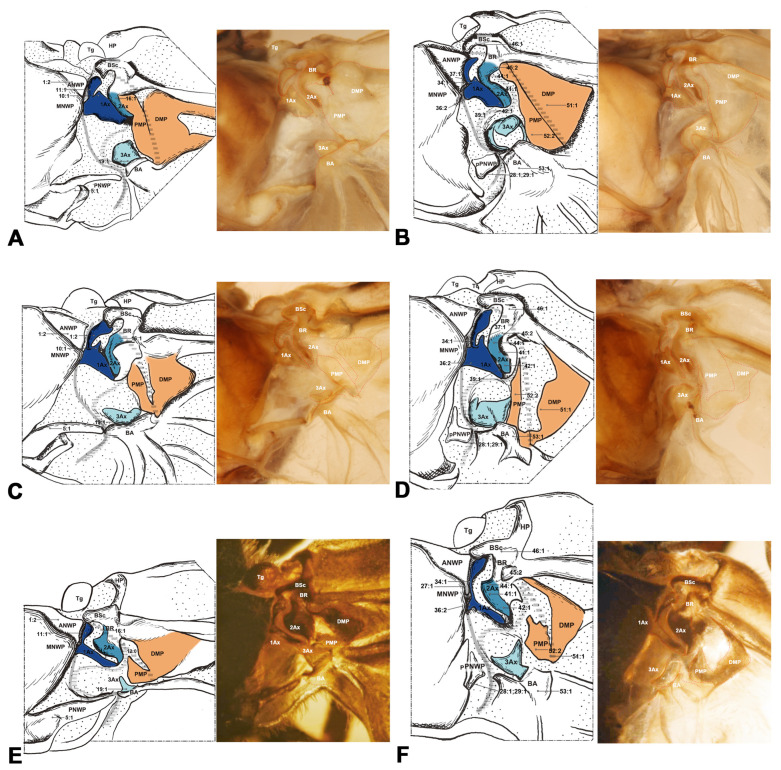
Wing base of Megaloptera. (**A**) *Protohermes costalis* Walker (Corydalinae), forewing base; (**B**) same, hindwing base; (**C**) *Neochauliodes punctatolosus* Liu & Yang (Chauliodinae), forewing base; (**D**) same, hindwing base; (**E**)*Sialis sibirica* McLachlan (Sialidae), forewing base; (**F**) same, hindwing base.

**Figure 7:**
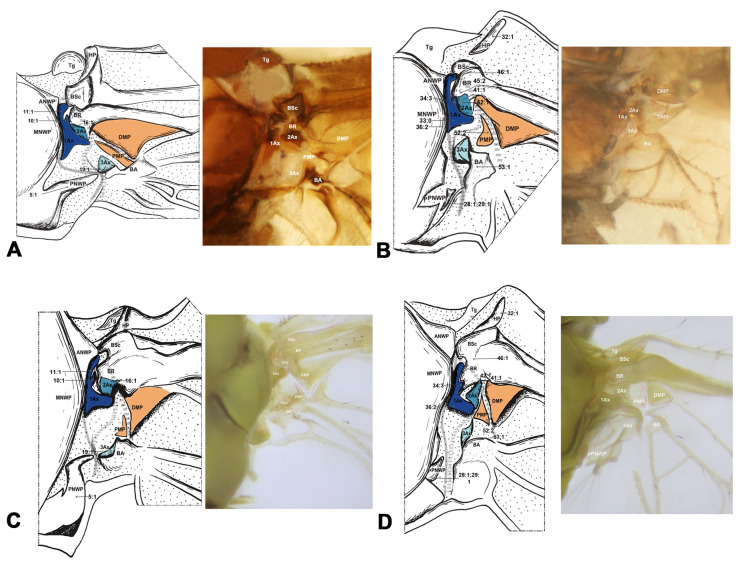
Wing base of Neuroptera. (**A**) *Heterosmylus wolonganus* Yang (Osmylidae), forewing base; (**B**) same, hindwing base; (**C**) *Chrysoperla* sp. (Chrysopidae), forewing base; (**D**) same, hindwing base.

**Figure 8:**
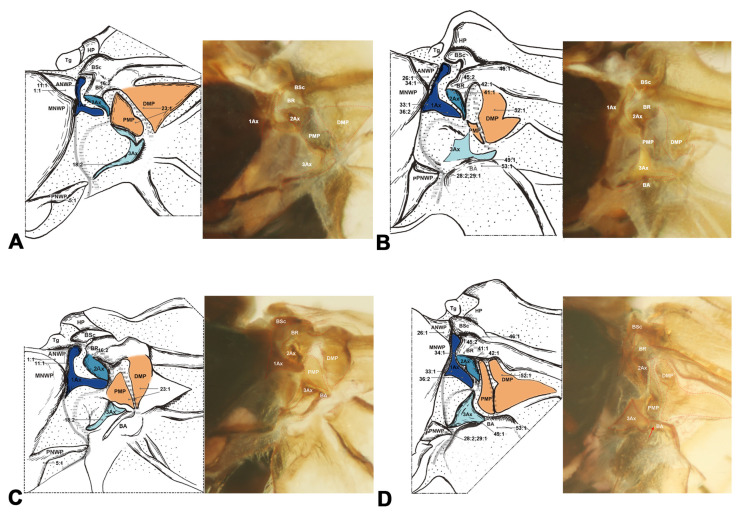
Wing base of Raphidioptera. (**A**) *Xanthostigma gobicola* Aspöck & Aspöck (Raphidiidae), forewing base; (**B**) same, hindwing base; (**C**) *Inocellia fujiana* Yang (Inocelliidae), forewing base; (**D**) same, hindwing base.

### 3.2. Character Description of Wing Base Structures Used for Phylogenetic Analysis

#### 3.2.1. Forewing

1. Shape of ANWP: (0) not triangular, stripe-like, or tubular-like; (1) nearly acute triangular; (2) nearly obtuse triangular; (3) stripe-like; (4) tubular-like. State 1 appeared in Raphidioptera (Figure 8A,C), and state 2 appeared in Megaloptera (Figure 6A,C,E). In Hymenoptera, the tubular-like ANWP of the mesonotum is obviously different from that of other orders in Holometabola (Figure 2A,C,E). (CI = 1, RI = 1).
2. Position of ANWP: (0) not anterior to Tg; (1) anterior to Tg. State 1 appeared in Hymenoptera (Figure 2A,C,E). (CI = 1, RI = 1).
3. Posterodistal projection of ANWP: (0) absent; (1) present. State 1 appeared in outgroups, Trichoptera (Figure 3A), and Lepidoptera (Figure 3C,E). (CI = 0.5, RI = 0.75).
4. Anterodistal projection of MNWP: (0) absent; (1) present. State 1 appeared exclusively in Trichoptera (Figure 3A), which might indicate a unique evolutionary characteristic of this order. (CI = 1, RI = 1).
5. Shape of PNWP: (0) not as for state (1) or (2); (1) long and slender process, at least 2 times longer than wide; (2) vestigial and indistinct. State 1 appeared in Trichoptera, Lepidoptera, Mecoptera, Coleoptera, Megaloptera, Neuroptera, and Raphidioptera (Figure 3A,C,E, Figure 4A, Figure 5A,C,D, Figure 7A,C, and Figure 8A,C). State 2 appeared in Xyelidae of Hymenoptera (Figure 2A), Diptera (Figure 4C–F), and Strepsiptera. (CI = 0.4, RI = 0.7).
6. Relationship between Tg and other sclerites: (0) Tg does not cover any sclerite; (1) Tg covers BSc or more; (2) Tg is absent. State 1 appeared in some Hymenoptera and Trichoptera (Figure 2C,E). The tegula is absent in Coleoptera, a phenomenon that may be closely related to the unique elytra of the forewing. (CI = 0.667, RI = 0.857).
7. Shape of Tg: (0) circular; (1) triangular; (2) rectangular; (3) absent. State 1 appeared in Lepidoptera. State 2 appeared in Mecoptera (Figure 4A). State 3 appeared in Coleoptera. (CI = 1, RI = 1).
8. 1Ax: (0) individual sclerite; (1) fused to notum or absent. State 1 appeared in some Coleoptera, such as Cupedidae and Melolonthidae. (CI = 0.5, RI = 0.5).
9. Shape of 1Ax: (0) not strongly swelling; (1) strongly swelling. State 1 appeared exclusively in Hymenoptera (Figure 2A,C,E), and it may be the autapomorphy of this order. (CI = 1, RI = 1).
10. Neck of 1Ax: (0) without projection, distal margin concave; (1) with a projection. State 1 appeared in Trichoptera, Lepidoptera, some Neuroptera, and some Megaloptera (Figure 3A,C,E, Figure 6A,C, and Figure 7A,C). (CI = 0.333, RI = 0.714).
11. Change in width from neck to head of 1Ax: (0) unchanged; (1) widened apically in head, continuous; (2) thinned apically in head, continuous; (3) thinned in boundary, widened in head, discontinuous. State 1 appeared in Neuroptera, Megaloptera, Raphidioptera, Coleoptera, Trichoptera, and Strepsiptera (Figure 3A, Figure 6A,C,E, Figure 7A,C, and Figure 8A,C). State 2 appeared in Lepidoptera (Figure 3C,E). State 3 appeared in Mecoptera and Diptera (Figure 4A,C–F). (CI = 1, RI = 1).
12. Shape of body of 1Ax: (0) nearly triangular; (1) nearly rectangular, proximal lobe as long as distal lobe; (2) heel-like. State 1 appeared in Trichoptera and Lepidoptera (Figure 3A,C,E). State 2 appeared in Diptera and Strepsiptera (Figure 4C–F). (CI = 0.667, RI = 0.833).
13. Anteroproximal part of the body of 1Ax: (0) without projection; (1) with a projection. State 1 appeared in Trichoptera, Lepidoptera, Mecoptera, and Diptera (Figure 3A,C,E and Figure 4A,C–F). (CI = 1, RI = 1).
14. Anterodistal part of the body of 1Ax: (0) without projection; (1) with a projection. State 1 appeared in Hymenoptera (Figure 2A,C,E), which may be an autapomorphy of this order. (CI = 1, RI = 1).
15. Angle between distal margins of body and neck of 1Ax: (0) less 120°; (1) 120°–180°; (2) 180°. State 1 appeared in some Neuroptera, Hymenoptera, Trichoptera, and Diptera (Figure 2A,C,E, Figure 3A, Figure 4A,C–F, and Figure 7A). (CI = 0.5, RI = 0.8).
16. Interaction between BR and 2Ax: (0) BR not fused to 2Ax, membranous insertion present; (1) BR approximately as wide as 2Ax and fused to it; (2) BR approximately half as wide as 2Ax and fused to it; (3) BR connected to 2Ax by a narrow, sclerotized stripe. State 1 appeared in Neuroptera, Megaloptera, Hymenoptera, Trichoptera, Lepidoptera, Mecoptera, and some Diptera (Figure 2A,C,E, Figure 3A,C,E, Figure 4A,C,D, Figure 6A,C,E, and Figure 7A,C). State 2 appeared in Raphidioptera (Figure 8A,C), and state 3 appeared in some Coleoptera. (CI = 1, RI = 1).
17. Number of lobes of 3Ax: (0) 3; (1) 2. State 1 appeared in Hymenoptera (Figure 2A,C,E). (CI = 1, RI = 1).
18. Shape of 3Ax: (0) plate-like; (1) slender proximal lobe; (2) slender proximal and anterior or distal lobe; (3) stripe-like. State 1 appeared in Trichoptera, Lepidoptera, Mecoptera, and Diptera (Figure 3A,C,E and Figure 4A,C–F). State 2 appeared in Raphidioptera (Figure 8A,C). State 3 appeared in Hymenoptera (Figure 2A,C,E). (CI = 1, RI = 1).
19. Contact between 3Ax and BA: (0) separated; (1) partly fused. State 1 appeared in Hymenoptera, Neuroptera, Megaloptera, Trichoptera, Lepidoptera, Mecoptera, and Diptera (Figure 2A,C,E, Figure 3A,C,E, Figure 4A,C–F, Figure 6A,C,E and Figure 7A,C). (CI = 0.333, RI = 0.778).
20. BR and DMP: (0) separated; (1) fused to a plate; (2) absent. State 1 appeared in Lepidoptera (Figure 3C,E). State 2 appeared in Strepsiptera. (CI = 1, RI = 1).
21. Size of DMP: (0) distinctly larger than 1Ax; (1) about as large as 1Ax; (2) smaller than 1Ax. State 1 appeared in Trichoptera, Lepidoptera, Mecoptera, and Diptera (Figure 3A,C,E and Figure 4A,C–F). State 2 appeared in Hymenoptera (Figure 2A,C,E). (CI = 1, RI = 1).
22. DMP: (0) not strongly swelling; (1) strongly swelling; (2) absent. State 1 appeared in Hymenoptera (Figure 2A,C,E). In Strepsiptera, the DMP was absent. (CI = 0.667, RI = 0).
23. DMP and PMP: (0) both sclerotized, but less than 1Ax; (1) both less sclerotized; (2) both as sclerotized as 1Ax; (3) DMP distinctly more sclerotized than PMP; (4) absent. State 1 appeared in Raphidioptera (Figure 6A,C). State 2 appeared in Trichoptera, Lepidoptera, Mecoptera, and Diptera (Figure 3A,C,E and Figure 4A,C–F). State 3 appeared in Hymenoptera (Figure 2A,C,E). In Strepsiptera, the median plates were absent. (CI = 1, RI = 1).
24. BA: (0) as large as or smaller than 3Ax; (1) 2 times larger than 3Ax. State 1 appeared in Mecoptera and Diptera (Figure 4A,C–F). (CI = 1, RI = 1).
25. Shape of BA: (0) different from 3Ax; (1) almost the same as 3Ax. State 1 appeared in Hymenoptera (Figure 2A,C,E). (CI = 1, RI = 1).

#### 3.2.2. Hindwing

**Figure 9:**
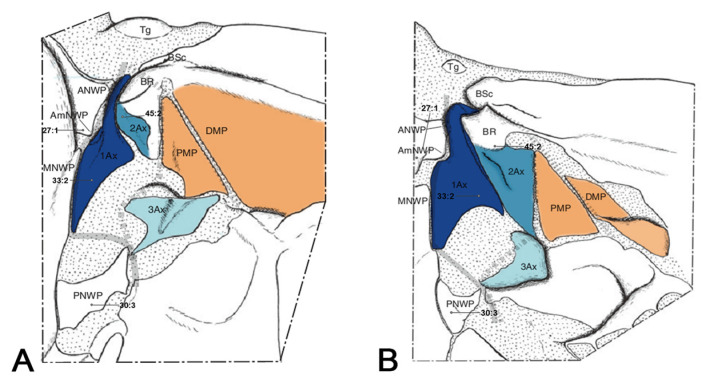
Wing base of outgroups. (**A**) *Illiesoperla australis* (Plecoptera: Gripopterygidae), hindwing base [59]; (**B**) *Homorocoryphus ineosus* (Orthoptera: Tettigoniidae), hindwing base [59].

26. ANWP configuration: (0) neither triangular nor stripe-like; (1) nearly triangular; (2) stripe-like, directed posteriorly; (3) stripe-like, directed anteriorly. State 1 appeared in some Raphidioptera, Coleoptera, Hymenoptera, and Strepsiptera (Figure 2B,D,F, Figure 5A,D, and Figure 8B,D). State 2 appeared in Neuroptera and Megaloptera. (Figure 6B,D,F and Figure 7B,D). (CI = 0.6, RI = 0.875).
27. Posterodistal projection of ANWP: (0) absent; (1) present. State 1 appeared in some Megaloptera, Trichoptera, Mecoptera, and some outgroups. (Figure 3B, Figure 6F, and Figure 9A,B). (CI = 0.25, RI = 0.25).
28. Shape of pPNWP: (0) absent; (1) neither U-shaped, triangular, or stripe-like; (2) triangular; (3) U-shaped; (4) present, stripe-like. State 1 appeared in Neuroptera and Megaloptera (Figure 6B,D,F and Figure 7B,D). State 2 appeared in Raphidioptera (Figure 8B,D). State 3 appeared in Mecoptera (Figure 4B). State 4 appeared in some Hymenoptera (Figure 2B). (CI = 1, RI = 1).
29. pPNWP: (0) absent; (1) sclerotized; (2) membranous. State 1 appeared in Neuroptera, Raphidioptera, and Megaloptera (Figure 6B,D,F, Figure 7B,D and Figure 8B,D). State 2 appeared in Hymenoptera and Mecoptera (Figure 2B and Figure 4B). (CI = 0.667, RI = 0.889).
30. Shape of PNWP: (0) shorter than twice its width; (1) roughly twice as long as its width; (2) roughly three times as long as its width; (3) vestigial. State 1 appeared in Hymenoptera (Figure 2B,D,F). State 2 appeared in Coleoptera, Trichoptera, and Lepidoptera (Figure 3B,D,F and Figure 5A–D). State 3 appeared in Mecoptera, Diptera, and outgroups (Figure 4B and Figure 9A,B). (CI = 0.75, RI = 0.938).
31. Tg: (0) present; (1) absent. State 1 appeared in Lepidoptera and Coleoptera (Figure 3D,F and Figure 5A–D). In our observation, the Tg of the hindwing in Neuroptera was only membranous, not a distinctly sclerotized structure. (CI = 0.5, RI = 0.833).
32. HP: (0) fused to end of the costal vein; (1) separated from the costal vein by a membrane; (2) either missing or fused to the costal vein in a manner that is difficult to distinguish. State 1 appeared in some Coleoptera, Mecoptera, and Neuroptera (Figure 4B, Figure 5A–C, and Figure 7B,D). State 2 appeared in Strepsiptera. (CI = 0.4, RI = 0.727).
33. Length ratio of 1Ax and metanotum: (0) notum more than 3.5 times longer; (1) notum 3–3.5 times longer; (2) notum 2.4–3 times longer; (3) notum 1.4–2.4 times longer. State 1 appeared in Raphidioptera and some Hymenoptera (Figure 2D,F and Figure 8B,D). State 2 appeared in outgroups, Trichoptera, and Lepidoptera (Figure 3B,D,F and Figure 9A,B). (CI = 0.6, RI = 0.818). State 3 appeared in Coleoptera (Figure 5A–D).
34. Neck of 1Ax: (0) narrower than head region, with a straight distal margin; (1) narrower than the head region, with a concave distal margin; (2) about as wide as the indistinct head region; (3) projection at the distal margin; (4) extremely short or absent. State 1 appeared in Raphidioptera, Megaloptera, Coleoptera, Lepidoptera, Mecoptera, and Strepsiptera (Figure 3D,F, Figure 4B, Figure 5A–D, Figure 6B,D, and Figure 8B,D). State 2 appeared in some Diptera. State 3 appeared in some Neuroptera and Trichoptera (Figure 3B and Figure 7B,D). State 4 appeared in all Hymenoptera (Figure 2B,D,F), but in Tenthredinidae and Diprionidae, the head of 1Ax was present, differing from Beutel et al. [17] who reported the head of the hindwing 1Ax as absent. (CI = 0.667, RI = 0.750).
35. Neck of 1Ax: (0) present; (1) absent. State 1 appeared in Hymenoptera (Figure 2B,D,F). (CI = 1, RI = 1).
36. Proximal lobe of the body of 1Ax: (0) distinctly longer than the distal lobe; (1) as long as the distal lobe; (2) shorter than the distal lobe. State 1 appeared in some Coleoptera, Hymenoptera, some Mecoptera, and Strepsiptera (Figure 2B,D,F, Figure 4B, and Figure 5B–D). State 2 appeared in Neuroptera, Megaloptera, Raphidioptera, Trichoptera, Lepidoptera, and some Mecoptera (Figure 3B,D,F, Figure 6B,D,F, Figure 7B,D, and Figure 8B,D). (CI = 0.333, RI = 0.636).
37. Anterodistal part of the body of 1Ax: (0) without projection; (1) with a projection. State 1 appeared in some Megaloptera (Figure 6B,D). (CI = 1, RI = 1).
38. Angle between the neck and the body of 1Ax: (0) wider than 130°; (1) less than 130°; (2) neck absent. State 1 appeared in Neuroptera, Megaloptera, Raphidioptera, Coleoptera, Trichoptera, Lepidoptera, some Diptera, and Strepsiptera (Figure 3B,D,F, Figure 5A–D, Figure 6B,D, Figure 7B,D, and Figure 8B,D). (CI = 0.667, RI = 0.8).
39. Contact between 2Ax and 1Ax: (0) proximo-cranial part of 2Ax articulates with 1Ax, proximo-caudal part separated from 1Ax by a membrane; (1) proximo-caudal part of 2Ax articulates with 1Ax, proximo-cranial part separated from 1Ax by a membrane; (2) articulation formed by proximo-cranial and proximo-caudal parts, each separated from 1Ax by a narrow membranous area. State 1 appeared in Megaloptera, Raphidioptera, Hymenoptera, Trichoptera, and some Mecoptera (Figure 2B,D,F, Figure 3B, Figure 5A–D, and Figure 6B,D). State 2 appeared in Lepidoptera (Figure 3D,F). (CI = 0.4, RI = 0.571).
40. Contact of 1Ax and 2Ax: (0) 1Ax does not cover 2Ax; (1) 1Ax covers 2Ax. (CI = 1, RI = 1). State 1 appeared in Coleoptera. (Figure 5B–D).
41. Shape of 2Ax: (0) not as in state (1) or state (2); (1) almost stripe-like but bends distally apically; (2) almost rectangular and does not bend. State 1 appeared in Neuroptera, Megaloptera, and Raphidioptera (Figure 6B,D,F, Figure 7B,D, and Figure 8B,D). State 2 appeared in Hymenoptera (Figure 2B,D,F). (CI = 1, RI = 1).
42. Size of 2Ax: (0) larger than the distal lobe of the body of 1Ax; (1) approximately equal in length to the distal lobe of 1Ax’s body. State 1 appeared in Hymenoptera, Neuroptera, Raphidioptera, Megaloptera, some Coleoptera, some Trichoptera, Lepidoptera, and Strepsiptera (Figure 2B, Figure 3B,D, Figure 5B–D, Figure 6B,D, Figure 7B,D, and Figure 8B,D). (CI = 0.167, RI = 0.375).
43. Proximal part of 2Ax: (0) not extending under the body of 1Ax; (1) extending under the body of 1Ax. State 1 appeared in some Coleoptera (Figure 5B–D). (CI = 1, RI = 1).
44. Anterior part of 2Ax: (0) bends proximally; (1) bends distally. State 1 appeared in Megaloptera (Figure 6B,D,F). (CI = 1, RI = 1).
45. Contact between BR and 2Ax: (0) separated; (1) connected by a narrow, sclerotized stripe; (2) fused directly. State 1 appeared in some Coleoptera (Figure 5A,D). State 2 appeared in Neuroptera, Raphidioptera, Megaloptera, Trichoptera, Lepidoptera, and Mecoptera (Figure 3B,D,F, Figure 4B, Figure 6B,D,F, Figure 7B,D, Figure 8B,D, and Figure 9A,B). (CI = 0.5, RI = 0.714).
46. BSc and BR: (0) separated or vestigial BR; (1) partly fused; (2) completely fused. State 1 appeared in Neuroptera, Megaloptera, and Raphidioptera (Figure 6B,D,F, Figure 7B,D, and Figure 8B,D). State 2 appeared in Hymenoptera (Figure 2B,D,F). (CI = 1, RI = 1).
47. 3Ax: (0) lacking a detached sclerite; (1) featuring a separate sclerite located between 1Ax and 3Ax. State 1 appeared in Coleoptera (Figure 5A–D). (CI = 1, RI = 1).
48. Detached part of 3Ax: (0) absent; (1) close to PMP; (2) not close to PMP. (CI = 1, RI = 1). State 1 appeared in Cupedidae (Figure 5A). State 2 appeared in other families of Coleoptera. (Figure 5B–D).
49. 3Ax and BA: (0) separated; (1) partly fused. State 1 appeared in Raphidioptera, Trichoptera, Lepidoptera, and Strepsiptera (Figure 3B,D,F and Figure 8B,D). (CI = 0.333, RI = 0.6).
50. Shape of 3Ax: (0) plate-like; (1) slender proximal lobe. State 1 appeared in Coleoptera (Figure 5A–D). (CI = 1, RI = 1).
51. DMP and PMP: (0) separated; (1) partly fused. State 1 appeared in Megaloptera, some Coleoptera, Lepidoptera, and Strepsiptera (Figure 3D,F, Figure 5A,C,D, and Figure 6B,D). (CI = 0.258, RI = 0.667).
52. PMP: (0) sclerotized; (1) almost membranous; (2) less sclerotized. State 1 appeared in Raphidioptera (Figure 8B,D), and state 2 appeared in Neuroptera and Megaloptera (Figure 6B,D,F and Figure 7B,D). (CI = 1, RI = 1).
53. Size of BA: (0) indistinguishable or not comparable; (1) smaller than 3Ax; (2) distinctly larger than 3Ax; (3) as large as 3Ax. State 1 appeared in Neuroptera, Raphidioptera, Megaloptera, Coleoptera, and Strepsiptera (Figure 5A–D, Figure 6B,D, Figure 7B,D, and Figure 8B,D). State 2 appeared in Hymenoptera (Figure 2B,D,F). (CI = 1, RI = 1).

### 3.3. Phylogenetic Analyses

**Figure 10:**
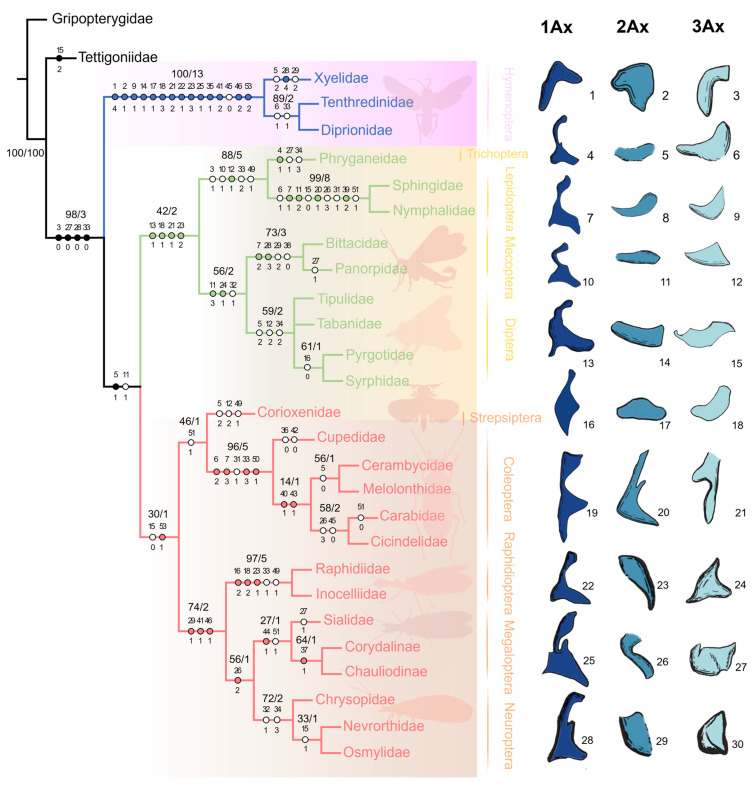
Phylogenetic tree of Holometabola based on wing base data. A strict consensus from parsimonious trees is displayed, focusing on forewing and hindwing bases. Features of clear mapping of unambiguous characters: filled circles for homologous traits, open circles for reversals or parallels. Character states are below the circles. Numbers on nodes indicate the bootstrap values and Bremer’s decay indices. The sclerites on the right side represent the 1Ax, 2Ax, and 3Ax of the respective orders. Sclerites numbered 1–12 and 16–30 are from the hindwing base, while sclerites numbered 13–15 are from the forewing base.

Our matrix analysis using the matrix method resulted in a single most parsimonious tree (MPT) (tree length = 146; CI = 0.66; RI = 0.85). The strict consensus tree is illustrated in Figure 10. Furthermore, PAUP*4.0b10 confirmed identical findings.

The monophyly of Holometabola was well supported by the following four homologous apomorphies: (1) the absence of posterodistal projection of the forewing ANWP (char. 3:0), (2) the absence of posterodistal projection of the hindwing ANWP (char. 27:0), (3) the absence of pPNWP (char. 28:0), and (4) a length ratio of the metanotum to the 1Ax of more than 3.5 times (char. 33:0).

In the study, Hymenoptera was recovered as the sister group to the remaining Holometabola orders, and its monophyly was corroborated by the following 14 homologous synapomorphies: (1) a tubular-like ANWP (char. 1:4), (2) an anterior position of the ANWP relative to that of the Tg (char. 2:1), (3) a strongly swollen 1Ax (char. 9:1), (4) projection of the anterodistal part of the body of 1Ax (char. 14:1), (5) two lobes of 3Ax (char. 17:1), (6) a stripe-like 3Ax (char. 18:3), (7) a DMP smaller than 1Ax (char. 21:2), (8) a strongly swollen DMP (char. 22:1), (9) a distinctly more sclerotized DMP than PMP (char. 23:3), (10) a BA and 3Ax with almost the same shape (char. 25:1), (11) the absence of the neck of the hindwing 1Ax (char. 35:1), (12) an almost rectangular 2Ax (char. 41:2), (13) completely fused BSc and BR (char. 46:2), and (14) a distinctly larger BA than 3Ax in the hindwing (char. 53:2).

In the remaining orders, excluding Hymenoptera of Holometabola, a sister relationship between Antliophora (Diptera, Mecoptera) and Amphiesmenoptera (Lepidoptera, Trichoptera) is supported by the following four homologous synapomorphies: (1) an anteroproximal part of the body of 1Ax with a projection (char. 13:1), (2) a slender proximal lobe of 3Ax (char. 18:1), (3) a DMP about as large as 1Ax (char. 21:1), and (4) both DMP and PMP sclerotized as much as 1Ax (char. 23:2).

Within the clade of Diptera and Mecoptera, there are two homologous synapomorphies that provide strong support for their sister-group relationship, as follows: (1) the transition in width from neck to head of 1Ax is characterized by a thinned boundary and a widened head apex in the forewing (char. 11:3), and (2) BA is twice as large as 3Ax (char. 24:1). Furthermore, the monophyly of Mecoptera received support from the following two additional homologous synapomorphies: (1) the shape of the Tg is rectangular (char. 7:2), and (2) the pPNWP is U-shaped (char. 28:3).

For the clade of Amphiesmenoptera (Lepidoptera, Trichoptera), there is one homologous synapomorphy that supports the monophyly. The body of the 1Ax is nearly rectangular, with a proximal lobe as long as the distal lobe (char. 12:1). The monophyly of Lepidoptera also received support from the following four homologous synapomorphies: (1) a triangular shape of Tg (char. 7:1), (2) a continuously apically thinned change in width from the neck to the head of 1Ax (char. 11:2), (3) BR and DMP fused to a plate (char. 20:1), and (4) 2Ax and 1Ax articulation formed by the proximo-cranial and proximo-caudal parts, both separated by a narrow membranous area (char. 39:2). As for Trichoptera, the monophyly was supported by one homologous synapomorphy, namely the anterodistal projection of MNWP (char. 4:1).

The sister relationship of Coleopterida and Neuropterida was supported by one homologous synapomorphy, namely a BA smaller than 3Ax (char. 53:1). Within this clade, one nonhomologous character supports the monophyly of Coleopterida, namely that 3Ax and BA are partly fused (char. 51:1). However, the monophyly of Coleoptera received support from the following four homologous synapomorphies: (1) the absence of a relationship between the Tg and other sclerites (char. 6:2), (2) a lack of Tg shape owing to its absence (char. 7:3), (3) a metanotum 1.4–2.4 times longer than 1Ax (char. 33:3), and (4) a slender proximal lobe of 3Ax (char. 50:1).

For the clade of Neuropterida (Neuroptera, Megaloptera, Raphidioptera), its monophyly was supported by the following three homologous synapomorphies: (1) the pPNWP is sclerotized (char. 29:1), (2) the shape of 2Ax is almost stripe-like but bends apically distally (char. 41:1), and (3) the BSc and BR are partly fused (char. 46:1). The sister relationship of Neuroptera and Megaloptera received support from one homologous synapomorphy, namely the shape of ANWP is stripe-like and directed posteriorly (char. 26:2). The monophyly of Raphidioptera received support from the following three homologous synapomorphies: (1) a BR approximately half as wide as 2Ax and fused to it (char. 16:2), (2) slender proximal and either anterior or distal lobes of 3Ax (char. 18:2), and (3) less sclerotized DMP and PMP (char. 23:1).

## 4. Discussion

Our phylogenetic analysis of forewing and hindwing base structural data supports the monophyly of Holometabola with a 98% bootstrap value and Bremer’s decay indices equal to 3. Our investigation is consistent with multiple studies relying on morphology and significant genome data [6,14,17,28]. Previous investigations on wing base morphology, largely conducted by Yoshizawa, were primarily centered on groups exhibiting incomplete metamorphosis [56,58,59,67]. Regarding Holometabola, wing base structure data are available in Beutel et al. [17] and Hörnschemeyer [6]. However, Beutel did not provide a detailed comparative morphological study of the selected wing base structures, while Hörnschemeyer used fewer representative families and species compared to our study. Beutel et al. [17] suggested three synapomorphies based on wing base structure to support the monophyly of Holometabola. In the forewing, the 1Ax articulates with the whole tail of 2Ax (char. 253:0). The distal lobe of the 3Ax of the forewing is identifiable (char. 257:0). The ANWP of the hindwing is almost triangular (char. 257:0).

As shown in Figure 10, Hymenoptera was assigned as the sister group to the remaining orders, a result consistent with the viewpoint proposed by many scholars [7,8,9,10,11]. The notion that Mecopterida (Antliophora + Amphiesmenoptera) forms a sister group with Neuropteroidea (Coleopterida + Neuropterida) aligns with the findings of Peters et al. [28], who recently analyzed the transcriptome and morphological data of complete metamorphosis insects. Our analyses are incongruent with the prevalent view that Hymenoptera is a sister group to Mecopterida and seriously challenge the argument about the sister relationship between Hymenoptera and Neuropterida, which is based on 18S rDNA sequence analysis [14,25,30]. There were significant differences observed in wing base structure between Hymenoptera and other orders. The apomorphy features supporting the monophyly of Hymenoptera are mostly unique. The most compelling evidence includes the absence of a neck in the 1Ax on the hindwing, the pronounced swelling of the 1Ax, and the distinct shape of the 3Ax, all of which distinguish Hymenoptera from other orders. Additional specific characters are detailed in the Results section. Among Hymenoptera, Xyelidae occupy a basal phylogenetic position, while Tenthredinidae form a sister group with Diprionidae.

There are two homologous synapomorphies supporting the sister relationship of Mecopterida and Neuropteroidea in our study; however, relatively low bootstrap values and Bremer’s decay indices indicate that stronger evidence should be found. This sister-group relationship was corroborated by Beutel et al. [17] based on the following homologous synapomorphies: (1) a slender PNWP of the mesothorax (char. 244:0), (2) an angle between the distal margin of the metathorax and the 1Ax of the metathorax greater than 50° (char. 275:0). The characteristic of a slender PNWP in the mesothorax was also observed in our experiment. Additionally, the monophyly of Mecopterida has been confirmed by four homologous synapomorphies. Furthermore, Amphiesmenoptera (Lepidoptera + Trichoptera) and Antliophora (Mecoptera + Diptera) are considered a sister group, a view that aligns with traditional perspectives and was corroborated by Peters et al. [28] based on molecular data, although incongruent with the findings of Beutel et al. [17], who suggested Antliophora as a sister group with Coleopterida. The monophyly of Amphiesmenoptera is supported by high bootstrap values and Bremer’s decay indices in our results. The sister-group relationship between Mecoptera and Diptera is consistent with the findings of Beutel et al. [17] and Peters et al. [28], supported by two synapomorphies and Bremer’s decay indices of 2 in our study.

Coleopterida (Coleoptera + Strepsiptera) was placed as a sister group to Neuropterida (Megaloptera + Neuroptera + Raphidioptera) in our experiment, which is congruent with the traditional perspective based on respective morphological and molecular datasets [1,14,15,26,30,36,68,69]. However, the sister-group relationship between Coleopterida and Neuropterida is inconsistent with the finding of Beutel et al. [17], who proposed that Coleopterida and Antliophora have a sister relationship. Our phylogenetic analysis of fore- and hindwing base structural data supports the monophyly of Coleoptera with high credibility, containing the following two synapomorphies: the absence of a Tg (char. 6:2) and a 1Ax relatively longer than the metathorax notum (char. 33:3). However, many studies based on molecular data suggest that Coleoptera is paraphyletic [30,70,71]. Additionally, there is only one synapomorphy supporting the sister-group relationship between Strepsiptera and Coleoptera. The phylogenetic status of Strepsiptera has long been a disputed and unresolved issue. Recently, most molecular phylogenetic studies have advocated for Strepsiptera and Coleoptera as sister taxa [26,28,36,68], but morphological evidence is lacking. The sister relationship between Strepsiptera and Coleoptera has been advocated by Friedrich et al. [16] and Beutel et al. [17], but their morphological support was considered weak or moderate in a subsequent study [69].

Our phylogenetic analysis within Neuropterida confirms its monophyly and positions Raphidioptera as the sister group to (Megaloptera + Neuroptera). This aligns with findings from most prior molecular studies [22,28,34,35,36,41,42,43]. Furthermore, the monophyly of Megaloptera and its sister-group relationship with Neuroptera were corroborated by our wing base data, which are consistent with many results based on morphology, mitochondrial genomics, and transcriptome data [28,35,37,43,47,70,71,72,73,74,75], as well as a recent phylogenetic analysis utilizing an integrative phylogenomic approach [46]. However, this challenges the view that Megaloptera was never recovered as monophyletic and with Raphidioptera in a clade sister to Neuroptera, as proposed by Winterton et al. [38]. They conducted a comprehensive phylogenetic study of Neuropterida, utilizing morphology and multilocus DNA sequence data across all extant families of Neuroptera, Megaloptera, and Raphidioptera. Wang et al. [45] primarily investigated the impact of highly informative selected genes or more realistic phylogenetic models on the reconstruction of Neuropterida phylogeny. Their research consistently affirmed the monophyly of Raphidioptera, Megaloptera, and Neuropterida. However, the monophyly of Neuroptera was only obtained when analyzing genes with strong signals or using robust models, which partially aligns with our findings. Furthermore, our study confirmed the higher-level classification of Megaloptera, dividing it into Corydalidae and Sialidae. Within Corydalidae, both Corydalinae and Chauliodinae were included. Furthermore, in Neuropterida, we consider that the pPNWP is a part of the distal part of the PNWP, with an irregular shape.

## 5. Conclusions

The structure of the wing base is a valuable tool for reconstructing the phylogeny of Holometabola. Many published works on phylogenetics corroborate the value of wing base structure for resolving higher-level phylogenetic problems [58,59]. In our study, several phylogenetic relationships among Holometabola were successfully resolved. The basal location of Hymenoptera, as well as the sister-group relationships of Strepsiptera and Coleoptera, Mecoptera and Diptera, Trichoptera and Lepidoptera, and Neuropterida and Coleopterida were confirmed. However, the relationship between Mecoptera and Siphonaptera remains unresolved due to the deficiencies of the wing base for Siphonaptera. Additionally, our study provided clear insights into the relationships among the three orders of Neuropterida. However, our present study faced a limitation due to the inadequate number of informative characters for resolving phylogenies at the family level. Future comprehensive studies may benefit from the geometric morphometrics of these wing base sclerites, potentially enhancing the resolution of family-level phylogeny.

## Data Availability

The authors confirm that all data used to support the findings are freely accessible without any restrictions. All relevant data are presented in the paper.

## Supplementary Materials

The following supporting information can be downloaded at: https://www.mdpi.com/article/10.3390/insects15030199/s1, Table S1: Examined taxa. Table S2: Data matrix for the present phylogenetic analysis.
