# Supplementary material for: Comparative Morphology of the Wing Base Structure Illuminates Higher-Level Phylogeny of Holometabola
Source: Insects. 2024 Mar 16;15(3):199. doi: 10.3390/insects15030199 (PMC10970979; doi:10.3390/insects15030199)
Supplement: Supplementary file 1 [file insects-15-00199-s001.zip › Table S1. Taxa examined clean.pdf]

Table 1. Taxa examined.

Taxon	Specimen	Collection information
Megaloptera		
**Corydalidae**		
Corydalinae		
Protohermes costalis (Walker)	2♂	CHINA, Zhejiang, Lin-an, Tianmushan, (CAU)
Chauliodinae		
Neochauliodes punctatolosus Liu & Yang	2♂	CHINA, Yunnan, Xishuangbanna, Menglun, 2009.V.30, Xiushuai Yang (CAU).
**Sialidae**		
Sialis sibirica McLachlan	2♀	CHINA, Heilongjiang, Maoershan, 2011.VII.2, Junchao Wang (CAU).
Neuroptera		
**Osmylidae**		
Heterosmylus wolonganus Yang	1♂	CHINA, Henan, Songxian, Baiyunshan, 2008.III.15, Weihai Li (CAU).
Thyridosmylus sp.	1♀	CHINA, Tibet, Hanmi, 2011.VIII.6, Lihua Wang (CAU).
**Chrysopidae**		
Chrysoperla sp.	1♀	CHINA, Sichuan, Emeishan, 2012.VIII.20, Liang Wang (CAU).
Italochrysa sp.	1♀	CHINA, Sichuan, Emeishan, 2012.VIII.20, Liang Wang (CAU).
**Nevrorthidae**		
Nipponeurorthus fuscinervis (Nakahara)	1♀	JAPAN, Hokkaido，Honbetsu, Yusen-kyo，2013.VII.1, Xingyue Liu (CAU).
Raphidioptera		
**Raphidiidae**		
Xanthostigma gobicola Aspöck & Aspöck	2♂	CHINA, Beijing, Yanqing, Songshan, 2010.VI.3, Lihua Wang (CAU).
**Inocelliidae**		
Inocellia fujiana Yang	2♂	CHINA, Shanxi, Taigu, (CAU).
Hymenoptera		
**Xyelidae**		
Xyela sp.	1♀	CHINA, Hubei, Yichang, Shennongjia, Xiaolongtan, 2011. V. 24, Zejian Li (CAU).

**Tenthredinidae**		
Tenthredo sp.	2♀	CHINA, Neimenggu, Saihanwula, Dadonggou, 2013.VII.24, Xiumei Lu (CAU).
**Diprionidae**		
Neodiprion huizeensis Xiao & Zhou, 1984	1♀	CHINA, Yunnan, Huize, 2013, Maoling, Sheng (CAU).
Coleoptera		
**Cupedidae**		
Tenomerga sp.	1♀	CHINA, Guangxi, Maoershan, 2003. VII. 2, Shuwen An (CAU).
**Carabidae**		
Macrocheilus bensoni Hope, 1838	1♂	CHINA, Yunnan, Mangshi, Mukang, 2013. VII. 14, Xuankun Li (CAU).
Chlaenius bimaculatus lynx Chaudoir, 1856	1♂	CHINA, Yunnan, Mangshi, Mukang, 2013. VII. 14, Xuankun Li (CAU).
**Cicindelidae**		
Cylindera sp.	2♂+1♀	CHINA, Yunnan, Gongshan, Bingzhonglu, 2013. VII. 4, Xuankun Li (CAU).
**Cerambycidae**		
Trichoferus guerryi (Pic, 1915)	2♀	CHINA , Yunnan, Baoshan, Baihualing, 2013. VII. 17, Xuankun Li (CAU).
**Melolonthidae**		
Metabolus flavescens Brenske	2♀	CHINA, Beijing, Campus of China Agricultural University, 2014.III.26, Liang Wang (CAU).
Lepidoptera		
**Sphingidae**		
Macroglossum sp.	1♂	CHINA, Beijing, Baiwangshan, 2012, IX. 28, Liang Wang (CAU).
**Nymphalidae**		
Polygonia c-aureum (Linnaeus)	1♂	CHINA, Beijing, Wulingshan, Yumidi, 2013. IX. 18, Yuyu Wang (CAU).
Diptera		
**Tipulidae**		
Tipula (Nippotipula) sinica Alexander	1♂	CHINA, Shaanxi, Louguantai, 1962, VIII.18, Fasheng Li (CAU).
**Pyrgotidae**		

Apyrgota breviventris Shi, 1998	1♂	CHINA, Yunnan, Baoshan, Baihualing,<br>2013. VII. 8, Xuankun Li (CAU).
**Syrphidae**		
Episyrphus balteatus (De Geer, 1776)	1♂, 1♀	CHINA, Zhejiang, Qingliangfeng, Tianchi, 2012. V. 15 (CAU).
**Tabanidae**		
Tabanus sp.	1♀	CHINA, Shanxi, Zhongtiaoshan, Botanical Garden,<br>2013. VII. 25, Tingting Zhang (CAU).
Trichoptera		
**Phryganeidae**		
Eubasilissa regina (McLachlan, 1871)	1♀	CHINA, Tibet, Motuo, 2011, VIII. 18, Qingbin Zhan (CAU).
Mecoptera		
**Bittacidae**		
Bittacus sp. Latreille	1♀+ 1♂	CHINA, Shaanxi, Liubaxian, Guanghuashan, 2013. VIII. 20, Yuqiang Xi (CAU).
**Panorpidae**		
Panorpa pryeri McLachlan, 1875		**Yoshizawa**
Strepsiptera		
**Corioxenidae**		
Triozocera sp.	1♂	CHINA, Guizhou, Dushan, 1981. 7, Wangmao Chen, Binghua Chen (CAU).
Plecoptera		
**Gripopterygidae**		
Illiesoperla australis		**Yoshizawa**
Orthoptera		
Ensifera		
**Tettigoniidae**		
Homorocoryphus ineosus		
