# Supplementary material for: Comparative Morphology of the Wing Base Structure Illuminates Higher-Level Phylogeny of Holometabola
Source: Insects. 2024 Mar 16;15(3):199. doi: 10.3390/insects15030199 (PMC10970979; doi:10.3390/insects15030199)
Supplement: Supplementary file 1 [file insects-15-00199-s001.zip › Table S2. Data matrix for the present phylogenetic analysis.pdf]

Table 2. Data matrix for present phylogenetic analysis

Taxa	1	2	3	4	5	6	7	8	9	10	11	12	13	14	15	16	17	18	19	20	21	22	23	24	25
Gripopterygidae	0	0	1	0	0	0	0	0	0	0	0	0	0	0	1	1	0	0	0	0	0	0	0	0	0
Tettigoniidae	0	0	1	0	0	0	0	0	0	0	0	0	0	0	2	1	0	0	0	0	0	0	0	0	0
Corydalinae	2	0	0	0	1	0	0	0	0	1	1	0	0	0	0	1	0	0	1	0	0	0	0	0	0
Chauliodinae	2	0	0	0	1	0	0	0	0	1	1	0	0	0	0	1	0	0	1	0	0	0	0	0	0
Sialidae	2	0	0	0	1	0	0	0	0	0	1	0	0	0	0	1	0	0	1	0	0	0	0	0	0
Nevrorthidae	3	0	0	0	1	0	0	0	0	1	1	0	0	0	1	1	0	0	1	0	0	0	0	0	0
Osmylidae	3	0	0	0	1	0	0	0	0	1	1	0	0	0	1	1	0	0	1	0	0	0	0	0	0
Chrysopidae	3	0	0	0	1	0	0	0	0	1	1	0	0	0	0	1	0	0	1	0	0	0	0	0	0
Raphidiidae	1	0	0	0	1	0	0	0	0	0	1	0	0	0	0	2	0	2	0	0	0	0	1	0	0
Inocelliidae	1	0	0	0	1	0	0	0	0	0	1	0	0	0	0	2	0	2	0	0	0	0	1	0	0
Cupedidae	0	0	0	0	1	2	3	1	0	0	1	0	0	0	0	3	0	?	0	?	?	?	?	?	0
Carabidae	0	0	0	0	1	2	3	0	0	0	1	?	?	0	0	?	0	?	0	?	?	?	?	?	0
Cicindelidae	0	0	0	0	1	2	3	0	0	0	1	?	?	0	?	?	0	?	0	?	?	?	?	?	0
Cerambycidae	0	0	0	0	0	2	3	1	0	0	1	?	?	0	0	?	0	?	0	?	?	?	?	?	0
Melolonthidae	0	0	0	0	0	2	3	1	0	0	1	?	?	0	?	?	0	?	0	?	?	?	?	?	0
Xyelidae	4	1	0	0	2	0	0	0	1	0	0	0	0	1	1	1	1	3	1	0	2	1	3	0	1
Tenthredinidae	4	1	0	0	0	1	0	0	1	0	0	0	0	1	1	1	1	3	1	0	2	1	3	0	1
Diprionidae	4	1	0	0	0	1	0	0	1	0	0	0	0	1	1	1	1	3	1	0	2	1	3	0	1
Phryganeidae	0	0	1	1	1	0	0	0	0	1	1	1	1	0	1	1	0	1	1	0	1	0	2	0	0

Sphingidae	0	0	1	0	1	1	1	0	0	1	2	1	1	0	0	1	0	1	1	1	1	0	2	0	0
Nymphalidae	0	0	1	0	1	1	1	0	0	1	2	1	1	0	0	1	0	1	1	1	1	0	2	0	0
Bittacidae	0	0	0	0	1	0	2	0	0	0	3	0	1	0	1	1	0	1	1	0	1	0	2	1	0
Panorpidae	0	0	0	0	1	0	2	0	0	0	3	0	1	0	1	1	0	1	1	0	1	0	2	1	0
Tipulidae	0	0	0	0	2	0	0	0	0	0	3	2	1	0	1	1	0	1	1	0	1	0	2	1	0
Tabanidae	0	0	0	0	2	0	0	0	0	0	3	2	1	0	1	1	0	1	1	0	1	0	2	1	0
Pyrgotidae	0	0	0	0	2	0	0	0	0	0	3	2	1	0	1	0	0	1	1	0	1	0	2	1	0
Syrphidae	0	0	0	0	2	0	0	0	0	0	3	2	1	0	1	0	0	1	1	0	1	0	2	1	0
Corioxenidae	0	0	0	0	2	0	0	0	0	0	1	2	0	0	?	?	0	?	0	2	?	2	4	?	?

	26	27	28	29	30	31	32	33	34	35	36	37	38	39	40	41	42	43	44	45	46	47	48	49	50	51	52	53
Gripopterygidae	0	1	0	?	3	0	0	2	2	0	0	0	0	?	0	0	0	0	0	2	0	0	0	0	0	0	0	0
Tettigoniidae	0	1	0	?	3	0	0	2	0	0	0	0	0	?	0	0	0	0	0	2	0	0	0	0	0	0	0	0
Corydalinae	2	0	1	1	0	0	0	0	1	0	2	1	1	1	0	1	1	0	1	2	1	0	0	0	0	1	2	1
Chauliodinae	2	0	1	1	0	0	0	0	1	0	2	1	1	1	0	1	1	0	1	2	1	0	0	0	0	1	2	1
Sialidae	2	1	1	1	0	0	0	0	1	0	2	0	1	0	0	1	1	0	1	2	1	0	0	0	0	1	2	1
Nevrorthidae	2	0	1	1	0	0	1	0	3	0	2	0	1	?	0	1	1	0	0	2	1	0	0	0	0	0	2	1
Osmylidae	2	0	1	1	0	0	1	0	3	0	2	0	1	?	0	1	1	0	0	2	1	0	0	0	0	0	2	1
Chrysopidae	2	0	1	1	0	0	1	0	3	0	2	0	1	?	0	1	1	0	0	2	1	0	0	0	0	0	2	1
Raphidiidae	1	0	2	1	0	0	0	1	1	0	2	0	1	?	0	1	1	0	0	2	1	0	0	1	0	0	1	1
Inocelliidae	1	0	2	1	0	0	0	1	1	0	2	0	1	0	0	1	1	0	0	2	1	0	0	1	0	0	1	1
Cupedidae	1	0	0	0	2	1	1	3	1	0	0	0	1	1	0	0	0	0	0	1	0	1	1	0	1	1	0	1

Carabidae	3	0	0	0	2	1	1	3	1	0	1	0	1	1	1	0	1	1	0	0	0	1	2	0	1	0	0	1
Cicindelidae	3	0	0	0	2	1	1	3	1	0	1	0	1	1	1	0	1	1	0	0	0	1	2	0	1	1	0	1
Cerambycidae	1	0	0	0	2	1	0	3	1	0	1	0	1	1	1	0	1	1	0	1	0	1	2	0	1	1	0	1
Melolonthidae	1	0	0	0	2	1	0	3	1	0	1	0	1	1	1	0	1	1	0	1	0	1	2	0	1	1	0	1
Xyelidae	1	0	4	2	1	0	0	0	4	1	1	0	2	1	0	2	1	0	0	0	2	0	0	0	0	0	0	2
Tenthredinidae	1	0	0	0	1	0	0	1	4	1	1	0	2	1	0	2	0	0	0	0	2	0	0	0	0	0	0	2
Diprionidae	1	0	0	0	1	0	0	1	4	1	1	0	2	1	0	2	0	0	0	0	2	0	0	0	0	0	0	2
Phryganeidae	0	1	0	0	2	0	0	2	3	0	2	0	1	1	0	0	1	0	0	2	0	0	0	1	0	0	0	0
Sphingidae	3	0	0	0	2	1	0	2	1	0	2	0	1	2	0	0	1	0	0	2	0	0	0	1	0	1	0	0
Nymphalidae	3	0	0	0	2	1	0	2	1	0	2	0	1	2	0	0	0	0	0	2	0	0	0	1	0	1	0	0
Bittacidae	0	0	3	2	3	0	1	0	1	0	1	0	0	1	0	0	0	0	0	2	0	0	0	0	0	0	0	0
Panorpidae	0	1	3	2	3	0	1	0	1	0	2	0	0	0	0	0	0	0	0	2	0	0	0	0	0	0	0	0
Tipulidae	0	0	0	0	3	0	1	0	2	0	0	0	1	0	0	0	1	0	0	0	0	0	0	0	0	?	0	0
Tabanidae	0	0	0	0	3	0	1	0	2	0	?	0	?	0	0	0	0	0	0	?	0	0	0	0	0	?	0	0
Pyrgotidae	0	0	0	0	3	0	1	0	2	0	?	0	?	0	0	0	?	0	?	?	0	0	0	0	0	?	0	0
Syrphidae	0	0	0	0	3	0	1	0	?	0	?	0	?	0	0	0	?	0	?	?	0	0	0	0	0	?	0	0
Corioxenidae	1	0	0	0	?	0	2	0	1	0	1	0	1	?	0	0	1	?	0	?	0	?	?	1	0	1	0	1
